# Change of Body Weight and Macrophage Inhibitory Cytokine-1 during Chemotherapy in Advanced Gastric Cancer: What Is Their Clinical Significance?

**DOI:** 10.1371/journal.pone.0088553

**Published:** 2014-02-28

**Authors:** Zhihao Lu, Li Yang, Jingwei Yu, Ming Lu, Xiaotian Zhang, Jian Li, Jun zhou, Xicheng Wang, Jifang Gong, Jing Gao, Jie Li, Yan Li, Lin Shen

**Affiliations:** 1 Key laboratory of Carcinogenesis and Translational Research (Ministry of Education), Department of Gastrointestinal Medical Oncology, Peking University School of Oncology, Beijing Cancer Hospital & Institute, Beijing, China; 2 Department of Oncology, Zhangzhou Municipal Hospital, Zhangzhou, Fujian Province, China; Peking University Cancer Hospital, China

## Abstract

**Background:**

Weight loss in advanced gastric cancer (GC) has been widely acknowledged to be a predictor for poor survival. However, very few studies have investigated the weight loss that occurs during chemotherapy. Therefore, we focused on weight loss during chemotherapy in patients with advanced GC and investigated the concentrations of macrophage inhibitory cytokine-1 (MIC-1), which has been recognized as a probable etiological factor in anorexia and weight loss.

**Methods:**

We analyzed 384 patients with inoperable locally advanced or metastatic GC receiving first-line chemotherapy. Patients were assigned to one of two groups on the basis of their weight change during chemotherapy: >3% weight loss and ≤3% weight loss. Serum MIC-1 and C-reactive protein (CRP) concentrations were also assessed in these patients.

**Results:**

The >3% weight loss group had shorter overall survival (OS; 12.0 months *vs.* 17.5 months, *P* = 0.000) than the ≤3% weight loss group, and the survival rates improved if the weight loss was reversed during chemotherapy. Although the MIC-1 concentrations were not correlated with weight loss before (*P* = 0.156) or during chemotherapy (*P* = 0.164), it correlated significantly with the CRP concentration (*P* = 0.001). Furthermore, elevated MIC-1 concentrations before chemotherapy (*P* = 0.017) and increased MIC-1 concentrations during chemotherapy (*P* = 0.001) were both found to be predictors of poor OS.

**Conclusions:**

Changes in the body weight during chemotherapy could influence the prognosis in patients with advanced GC, and the MIC-1 might be a potential predictive and prognostic biomarker in those patients.

## Introduction

Weight loss is common in cancer patients, and this could be attributed to diminished dietary intake as well as increased energy expenditure mediated by metabolic alterations caused by the tumor [1]. Approximately 80% of patients with upper gastrointestinal cancers have substantial weight loss [2].

Previous studies have investigated the effects of weight loss before chemotherapy on the outcomes of advanced GC [3], [4]. The mechanism of weight loss during chemotherapy differs from that of weight loss before chemotherapy because of the role played by chemotherapy and chemotherapy-related toxicities on body weight. Since chemotherapy-related factors are temporary, we aimed to investigate whether the weight loss occurring during chemotherapy was a transient phenomenon and whether it had any clinical significance. A study on subjects with stage III epithelial ovarian carcinoma indicated that weight change during primary chemotherapy was a potential prognostic factor for OS [5]. It is unclear, however, if weight change during chemotherapy could potentially impact the survival of patients with advanced GC.

MIC-1, a divergent member of the transforming growth factor-β (TGF-β) superfamily that is produced by macrophages in response to activation [6], is present in the circulation of individuals with a normal range of 150–1,150 pg/ml [7], [8]. It is expressed at high concentrations during inflammation [9]. Secretion of high concentrations of MIC-1 has also been reported in several types of cancer [10]–[12], and the role of this factor has been widely studied, including its role in tumorigenicity in malignant melanoma [13], invasiveness of gastric cancer [14], and poor survival in colorectal cancer [15]. Recently, MIC-1 has been found to be involved in the regulation of appetite and energy storage [16], [17]. In a study involving a small group of patients with cachectic prostate cancer, serum MIC-1 concentrations were significantly associated with weight loss [17]. However, the clinical significance of MIC-1, especially changes in the MIC-1 concentration during chemotherapy in advanced GC patients remains to be understood.

Therefore, we aimed to determine whether weight loss occurring during chemotherapy influences treatment outcomes and if changes in the serum MIC-1 concentrations are correlated with the weight loss and survival in patients with advanced GC.

## Patients and Methods

### Ethics statement

This study was approved by the Medical Ethics Committee of Peking University Cancer Hospital (Beijing, China) and was performed according to the Declaration of Helsinki Principles. Written informed consents were obtained from all study participants for their information to be stored in the hospital database and used for future research.

### Patients and data collection

Detailed clinical data of the patients treated at the Gastrointestinal Oncology Department of the Peking University Cancer Hospital were recorded in a regularly updated electronic database. Eligibility criteria included: (1) chemotherapy-naïve patients with pathologically confirmed, inoperable locally advanced or metastatic GC, (2) patients who received first-line chemotherapy, and (3) patients with a life expectancy ≥3 months. All the patients provided written informed consents before receiving chemotherapy.

Whole blood samples were obtained prior to and at the end of first-line chemotherapy for analysis of MIC-1 and CRP concentrations in the serum. A healthy control cohort consisting of laboratory and hospital staff was recruited for comparative MIC-1 analysis (*n* = 129; 54 men and 75 women; median age, 44 years; range, 20–80 years). Exclusion criteria for the controls included recent weight change, any illness, or pregnancy.

Weight loss before chemotherapy was recorded by direct questioning of the patients during a preliminary face-to-face assessment by the doctor at their first visit. The patients were asked if they had lost any weight since their illness began. Those who reported weight loss were then asked if they knew their stable weight before the illness. The extent of weight loss before chemotherapy was calculated as a percentage by comparing the measured weight with the reported weight. The patients were weighed at each chemotherapy visit, and an experienced team of nurses recorded the data. All the patients were weighed on spring balance scales without shoes and while wearing the same type of patient gowns each time. The extent of weight change during chemotherapy was determined by comparing the pre-treatment body weight (W1) with the body weight at each chemotherapy visit (W2). A relative change in body weight was calculated as a percentage according to the following formula: (W2−W1)/W1×100%.

Toxicity was recorded according to the National Cancer Institution (NCI) Common Toxicity Criteria Version 3.0 (CTC 3.0) by direct questioning, physical examination, and laboratory tests. Objective response to treatment was classified using the Response Evaluation Criteria in Solid Tumors (RECIST 1.0) every 6 weeks. Progression-free survival (PFS) and overall survival (OS) were calculated from the date of the first visit to the date of disease progression and death, respectively.

### Serum MIC-1 concentrations

The serum MIC-1 concentrations (pg/ml) were determined using a sensitive in-house sandwich enzyme-linked immunosorbent assay (ELISA), as previously described [7], [18]. All the samples were assayed at least two times, and the coefficient of variation between the samples was <10%.

### Systemic inflammation

Systemic inflammation was determined by CRP. Routine laboratory measurements of CRP were carried out before first-line chemotherapy. The limit of detection of the CRP assay was <0.3 mg/l, with the upper limit of normal values being <10.0 mg/l. Over the range of measurements, the coefficients of variation for these methods were less than 5% as established by routine quality control. A CRP concentration of >10.0 mg/l was used to define an acute-phase protein response.

### Statistical methods

SPSS (version 13.0) statistical software was used for the statistical analyses. A receiver-operating characteristic (ROC) curve was constructed to determine the optimal sensitivity and specificity followed by the determination of the cut-off value for serum MIC-1 concentrations. Serum MIC-1 data were presented as box plots. Mild outliers (MIC-1 concentration >1.5 times the interquartile range (IQR) above the third quartile) were represented by circles. For visual clarity, the Y-axes were limited to a maximum MIC-1 concentration of 8,000 pg/ml. Univariate survival analysis was performed using the Kaplan–Meier method with the log rank test. Multivariate survival analysis was performed using a Cox regression model including all known prognostic variables. All *P* values were two-sided and a *P* value of <0.05 was considered significant.

## Results

### Patient characteristics

From April 2004 to September 2011, 384 patients were found eligible for the study. Of these, 96 (25.0%) had locally advanced disease and 50 (52.1%) received gastrectomy with extended lymph node dissection (D2) after first-line chemotherapy. The last date of follow-up was September 1, 2012, and 21 patients (5.5%) were lost to follow-up. At this time, 264 patients (68.8%) died and the median OS was 13.9 months. The detailed patient characteristics are listed in Table 1.

**Table 1:** Patient clinicopathological variables and weight loss during chemotherapy.

	Total *n* (%)	Weight loss >3% *n* (%)	Weight loss ≤3% *n* (%)	*P*-value
Number of patients	384 (100)	195 (50.8)	189 (49.2)	
Gender				
Male	273 (71.1)	135 (49.5)	138 (50.5)	
Female	111 (28.9)	60 (54.1)	51 (45.9)	0.413
Age [median (range)]	57 (19–79)	57 (19–79)	57 (22–79)	
≤65 years old	289 (75.3)	145 (50.2)	144 (49.8)	
>65 years old	95 (24.7)	50 (52.6)	45 (47.4)	0.678
KPS before chemotherapy				
≤80	148 (38.5)	76 (51.4)	72 (48.6)	
>80	236 (61.5)	119 (50.4)	117 (49.6)	0.860
Weight loss before chemotherapy				
≤5%	164 (44.6)	81 (49.4)	83 (50.6)	
>5%	204 (55.4)	107 (52.5)	97 (47.5)	0.559
Histological differentiation				
Well/moderately differentiated	89 (23.2)	39 (43.8)	50 (56.2)	
Poorly differentiated	295 (76.8)	156 (52.9)	139 (47.1)	0.134
Stage				
Locally advanced	96 (25.0)	32 (33.3)	64 (66.7)	
Metastatic disease	288 (75.0)	163 (56.6)	125 (43.4)	0.000
Liver and/or lung metastasis				
Yes	154 (40.1)	87 (56.5)	67 (43.5)	
No	230 (59.9)	108 (47.0)	122 (53.0)	0.067
Peritoneal metastasis				
Yes	85 (22.1)	47 (55.3)	38 (44.7)	
No	299 (77.9)	148 (49.5)	151(50.5)	0.346
Ascites				
Yes	31 (8.1)	17 (54.8)	14 (45.2)	
No	353 (91.9)	178 (50.4)	175 (49.6)	0.637
Chemotherapeutic regimens				
Taxane-containing regimens	188 (49.0)	85 (45.2)	103 (54.8)	
Platinum-containing regimens	162 (42.2)	88 (54.3)	74 (45.7)	
Others#	34 (8.8)	22 (64.7)	12 (35.3)	0.056
Gastrectomy before chemotherapy				
Yes	33 (8.6)	17 (51.5)	16 (48.5)	
No	351 (91.4)	178 (50.7)	173 (49.3)	0.930
MIC-1 before chemotherapy (pg/ml) [median (inter-quartile range)]	1372 (775–2249)	1472 (884–2337)	1236 (664–2226)	0.164
MIC-1 change during chemotherapy				
Increase (Increase >20%)	78 (58.6)	46 (59.0)	32 (41.0)	
Non-increase (Increase ≤20% or decrease)	55 (41.4)	24 (43.6)	31 (56.4)	0.081
Gastrointestinal toxicity				
Grade 0	124 (32.3)	48 (38.7)	76 (61.3)	
Grade 1–2	210 (54.7)	115 (54.8)	95 (45.2)	
Grade 3–4	50 (13.0)	32 (64.0)	18 (36.0)	0.002
Severe (grade 3/4) toxicities of all kinds				
Yes	151 (39.3)	90 (59.6)	61 (40.4)	
No	233 (60.7)	105 (45.1)	128 (54.9)	0.005
Objective response				
Yes (CR+PR)	155 (40.4)	76 (49.0)	79 (51.0)	
No (SD+PD)	229 (59.6)	119 (52.0)	110 (48.0)	0.573

# Others include: Oxaliplatin-based, Irinotecan-based and S1 based regimens.

Mann–Whitney U test.

CR, complete response; PR, partial response; SD, stable disease; PD, progression disease; KPS, Karnofsky performance score; MIC-1, macrophage inhibitory cytokine-1.

### Relationship of MIC-1 with weight loss and systemic inflammation

#### Weight loss

Of the 368 patients with data for weight change before chemotherapy, 244 (66.3%) patients had lost a median of 8.8% (interquartile range, 6.2–14.1%) of their body weight. Of the 384 patients with data for weight change during chemotherapy, 244 (63.5%) patients had lost a median of 5.5% (interquartile range, 3.5–8.4%) of their body weight.

#### MIC-1

**Figure 1:**
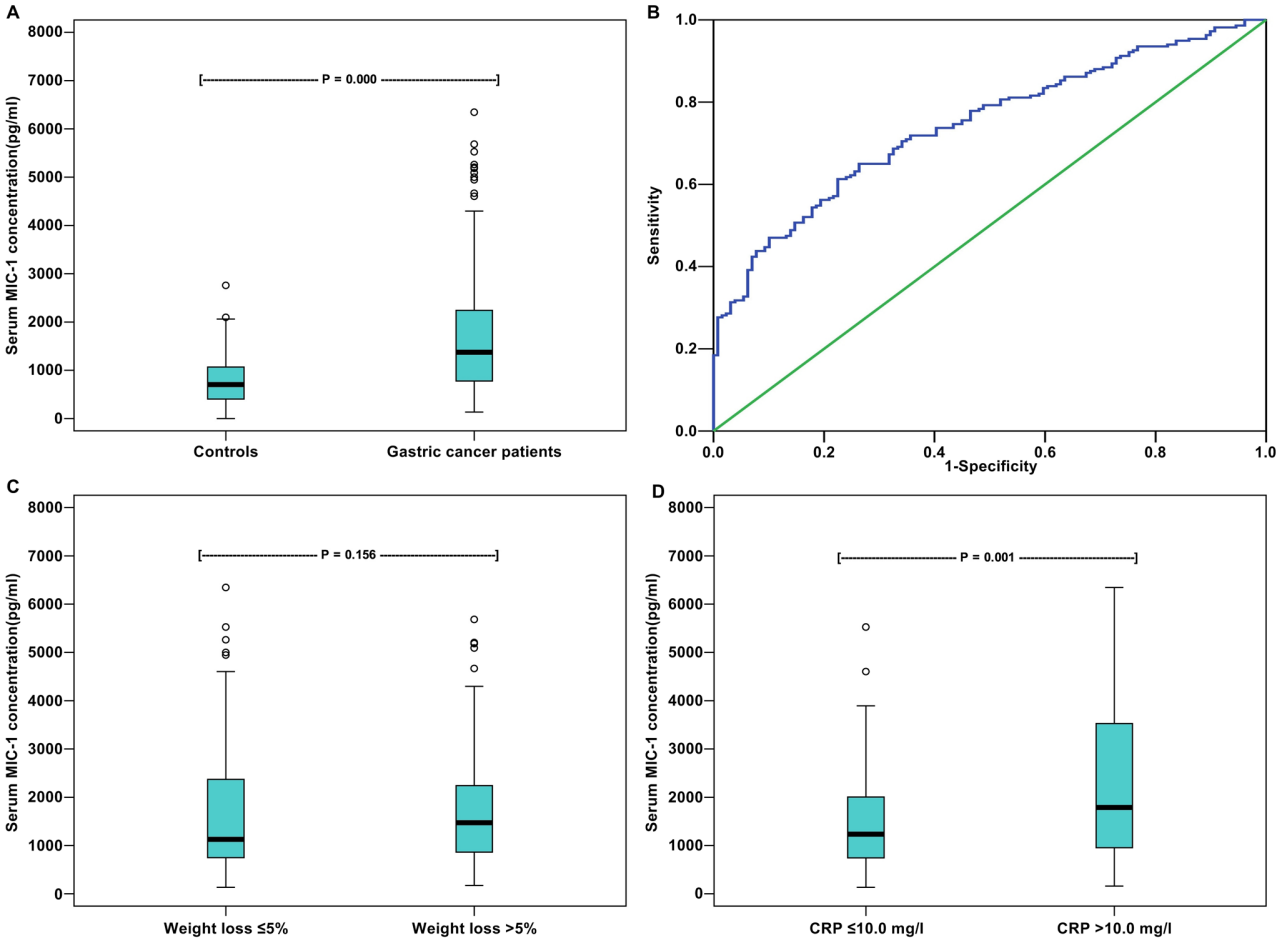
Serum MIC-1 concentrations in GC patients and ROC curve for determining MIC-1 cut-off value. A. Serum MIC-1 concentrations in GC patients (median  = 1372 pg/ml) and controls (median  = 351 pg/ml, *P* = 0.000); B. ROC curve for determining MIC-1 cut-off value (the area under the ROC curve: 0.743±0.026, *P* = 0.000); C. Serum MIC-1 concentrations in patients with >5% weight loss (median  = 1472 pg/ml) and ≤5% weight loss before chemotherapy (median  = 1128 pg/ml, *P* = 0.156); D. Serum MIC-1 concentrations in patients with CRP >10 mg/l (median  = 1787 pg/ml) and in patients with CRP ≤10 mg/l (median  = 1236 pg/ml, *P* = 0.001) before chemotherapy.

Blood samples of 217 patients were available for analysis of MIC-1 concentrations before chemotherapy. The serum MIC-1 concentrations were found to be elevated in patients with advanced GC when compared with the healthy controls (*P* = 0.000; Figure 1A). The MIC-1 values before chemotherapy correlated neither with histological differentiation (*P* = 0.722) nor with tumor stage (*P* = 0.439). Based on the results of the ROC analysis, the cut-off value was defined as 1120 pg/ml, and the sensitivity and specificity of the analysis was 61.3% and 77.5%, respectively (Figure 1B). At the end of the first-line chemotherapy, blood samples of 133 patients were obtained for analysis of MIC-1 concentrations. Patients were assigned to two groups on the basis of change of their MIC-1 concentrations: increase group (MIC-1 increase of >20%; 78 patients, 58.6%) and non-increase group (MIC-1 increase of ≤20% or decrease; 55 patients, 41.4%). (Table 1).

#### MIC-1 and weight loss

The serum MIC-1 concentrations did not correlate with weight loss before chemotherapy (*P* = 0.156; Figure 1C). Although the ratio of MIC-1 increasing was higher in patients with >3% weight loss during chemotherapy than in patients with ≤3% weight loss (59.0% *vs.* 41.0%, *P* = 0.081; Table 1), the difference did not reach statistical significance.

#### MIC-1 and systemic inflammation

Of the 217 patients with serum CRP concentration data before chemotherapy, 66 (30.4%) had CRP >10.0 mg/l. The serum MIC-1 concentrations were higher in patients with CRP >10.0 mg/l than in patients with CRP ≤10.0 mg/l (*P* = 0.001; Figure 1D).

### Univariate and multivariate analyses of risk factors for OS

Univariate analysis showed that KPS before chemotherapy, histological differentiation, stage, peritoneal metastasis, chemotherapy cycles, objective response, gastrectomy after chemotherapy, weight loss during chemotherapy, weight loss before chemotherapy, MIC-1 before chemotherapy and MIC-1 change during chemotherapy were significant predictors for OS (Table 2). In Cox regression model, including age, KPS before chemotherapy, histological differentiation, stage, peritoneal metastasis, chemotherapy cycles, objective response, weight loss during chemotherapy and weight loss before chemotherapy, all variables except for age and peritoneal metastasis were independent risk factors for OS. When adding MIC-1 before chemotherapy and MIC-1 change during chemotherapy to the model, only data of 133 patients with MIC-1 values were analyzed and both MIC-1 before chemotherapy and MIC-1 change during chemotherapy were independent risk factors for OS (Table 3).

**Table 2:** Univariate analysis of clinical factors for overall survival.

Variables	Number of patient *n* (%)	Survival (months) (95% CI^a^)	*P*-value
Gender			
Male	273 (71.1)	13.0 (12.4–15.4)	
Female	111 (28.9)	14.5 (11.0–18.0)	0.673
Age			
>65 yrs	95 (24.7)	13.4 (12.2–14.7)	
≤65 yrs	289 (75.3)	14.4 (12.4–16.4)	0.811
KPS before chemotherapy			
≤80	148 (38.5)	12.0 (10.6–13.3)	
>80	236 (61.5)	16.1 (14.0–18.2)	0.000
Histological differentiation			
Poorly differentiated	295 (76.8)	13.2 (11.9–14.5)	
Well/moderately differentiated	89 (23.2)	16.7 (13.8–19.5)	0.037
Stage			
Locally advanced	96 (25.0%)	19.2 (12.5–25.7)	
Metastatic disease	288 (75.0%)	13.4 (12.0–14.7)	0.001
Liver and/or lung metastasis			
Yes	154 (40.1)	13.9 (12.3–15.5)	
No	230 (59.9)	14.4 (12.2–16.6)	0.322
Peritoneal metastasis			
Yes	85 (22.1)	11.5 (8.3–14.7)	
No	299 (77.9)	14.5 (12.6–16.4)	0.000
Chemotherapy cycles			
<4cycles	141 (36.7)	10.3 (8.3–12.3)	
≥4cycles	243 (63.3)	16.0 (14.1–18.0)	0.029
Objective response			
Yes (CR+PR)	155 (40.4)	17.5 (15.3–19.8)	
No (SD+PD)	229 (59.6)	12.5 (10.9–14.2)	0.000
Gastrectomy after chemotherapy			
Yes	50 (13.0)	24.7 (11.7–37.7)	
No	334 (87.0)	13.2 (12.2–14.2)	0.000
Gastrectomy before chemotherapy			
Yes	33 (8.6)	22.0 (19.0–25.1)	
No	351 (91.4)	13.3 (12.2–14.4)	0.076
Gastrointestinal toxicity of 3–4 grade			
Yes	50 (13.0)	13.2 (12.7–13.8)	
No	334 (87.0)	14.2 (12.2–16.2)	0.320
Toxicities of 3–4 grade of all kinds			
Yes	151 (39.3)	13.8 (12.5–15.2)	
No	233 (60.7)	14.5 (12.1–16.9)	0.175
Weight loss during chemotherapy			
>3%	195 (50.8)	12.0 (10.5–13.6)	
≤3%	189 (49.2)	17.5 (13.7–21.4)	0.000
Weight loss before chemotherapy			
>5%	204 (55.4)	11.8 (10.6–12.9)	
≤5%	164 (44.6)	17.9 (14.6–21.3)	0.000
MIC-1 before chemotherapy (pg/ml)			
>1120	129 (59.4)	11.9 (10.8–13.1)	
≤1120	88 (40.6)	16.7 (13.0–20.3)	0.015
MIC-1 change during chemotherapy			
Increase (Increase >20%)	78 (58.6)	13.0 (11.0–15.0)	
Non-increase (Increase ≤20% or decrease)	55 (41.4)	17.8 (11.8–23.7)	0.030

a CI, confidence interval; CR, complete response; PR, partial response; SD, stable disease;

PD, progression disease; KPS, Karnofsky performance score; MIC-1, macrophage inhibitory cytokine-1.

**Table 3:** Univariate and multivariate analyses of risk factors for overall survival.

Variables	Univariate		Multivariate	
	Hazard ratio (95% CI^a^)	*P*-value	Hazard ratio (95% CI)	*P*-value
Age (>65 yrs/≤65 yrs)	0.967 (0.732–1.277)	0.811		0.973
KPS before chemotherapy (≤80/>80)	1.605 (1.251–2.059)	0.000	1.528 (1.177–1.985)	0.001
Histological differentiation (poorly/well or moderately)	1.362 (1.018–1.824)	0.037	1.393 (1.027–1.889)	0.033
Stage (locally advanced/metastatic disease)	0.599 (0.446–0.804)	0.001	0.566 (0.404–0.792)	0.001
Peritoneal metastasis(yes/no)	1.723 (1.292–2.299)	0.000	1.363 (0.997–1.864)	0.052
Chemotherapy cycles(<4cycles/≥4cycles)	1.322 (1.028–1.699)	0.029	1.527 (1.172–1.990)	0.002
Objective response (yes/no)	0.647 (0.505–0.828)	0.000	0.759 (0.585–0.985)	0.038
Weight loss during chemotherapy (>3%/≤3%)	1.780 (1.392–2.275)	0.000	1.478 (1.137–1.921)	0.004
Weight loss before chemotherapy (>5%/≤5%)	1.649 (1.282–2.122)	0.000	1.590 (1.225–2.062)	0.000
MIC-1 before chemotherapy (pg/ml) (>1120/≤1120)	1.553 (1.085–2.224)	0.015	1.856 (1.116–3.088)#	0.017#
MIC-1 change during chemotherapy (increase/non-increase)	1.644 (1.043–2.590)	0.030	2.198 (1.354–3.568)#	0.001#

a CI, confidence interval.

KPS, Karnofsky performance score; MIC-1, macrophage inhibitory cytokine-1.

# The hazard ratio and 95% confidence interval of the variable were derived from the multivariate Cox hazard proportional model constructed in the 133 complete subjects with MIC-1 concentrations, which was adjusted by age, KPS before chemotherapy, histological differentiation, stage, peritoneal metastasis, chemotherapy cycles, objective response, weight loss before chemotherapy, weight loss during chemotherapy, MIC-1 before chemotherapy and MIC-1 change during chemotherapy.

### Effect of weight change during chemotherapy on PFS and OS

**Figure 2:**
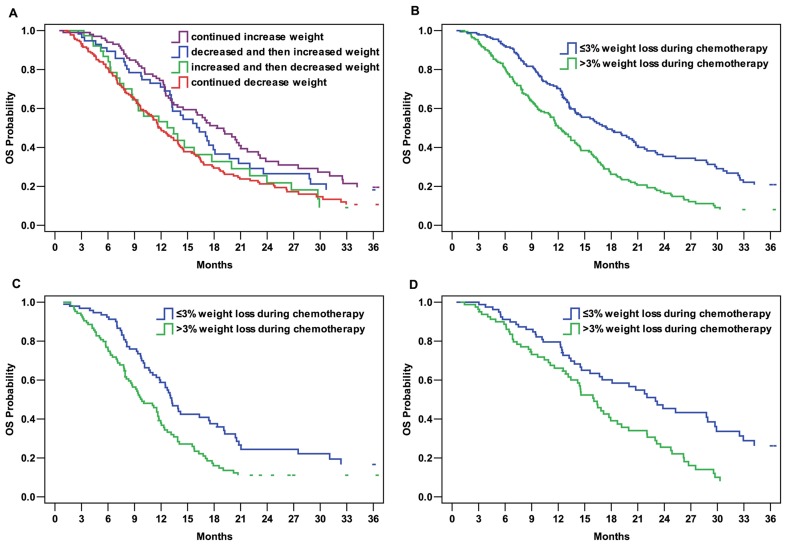
Kaplan–Meier curves of overall survival (OS) of patients according to weight change before or during chemotherapy. A. OS curve of patients grouped by weight change trends during chemotherapy (*P* = 0.003). B. OS curve of patients grouped by weight loss during chemotherapy (*P* = 0.000). C. OS curve of patients with >5% weight loss before chemotherapy grouped by weight loss during chemotherapy (*P* = 0.004). D. OS curve of patients with ≤5% weight loss before chemotherapy grouped by weight loss during chemotherapy (*P* = 0.001).

The 384 patients were classified into one of four groups on the basis of their trend in weight change during chemotherapy: group 1 included patients exhibiting a continued decrease in weight (*n* = 181); group 2 included patients whose weight first increased and then decreased (*n* = 39); group 3, patients whose weight first decreased and then increased (*n* = 57); and group 4, patients with increasing weight (*n* = 107). In the 334 patients who did not receive gastrectomy after chemotherapy, the median PFS were estimated to be 4.7, 5.4, 6.3, and 7.0 months in groups 1, 2, 3, and 4 respectively (*P* = 0.045). The median OS was estimated to be 11.8, 13.4, 16.3, and 18.4 months in groups 1, 2, 3, and 4 respectively (*P* = 0.003; Figure 2A).

During chemotherapy, patients with >3% weight loss had shorter PFS (5.2 months *vs.* 6.2 months; *P* = 0.032) than those with ≤3% weight loss. Similarly, of the 384 patients, patients with >3% weight loss had shorter OS (12.0 months *vs.* 17.5 months; *P* = 0.000; Figure 2B) than those with ≤3% weight loss. Furthermore, in the >5% weight loss before chemotherapy group, the patients with ≤3% weight loss during chemotherapy had longer OS compared to those with >3% weight loss (13.2 months *vs.* 9.8 months; *P* = 0.004; Figure 2C). This trend could also be found in the ≤5% weight loss before chemotherapy group (23.0 months *vs.* 16.0 months; *P* = 0.001; Figure 2D).

### Serum MIC-1 concentrations and prognosis

**Figure 3:**
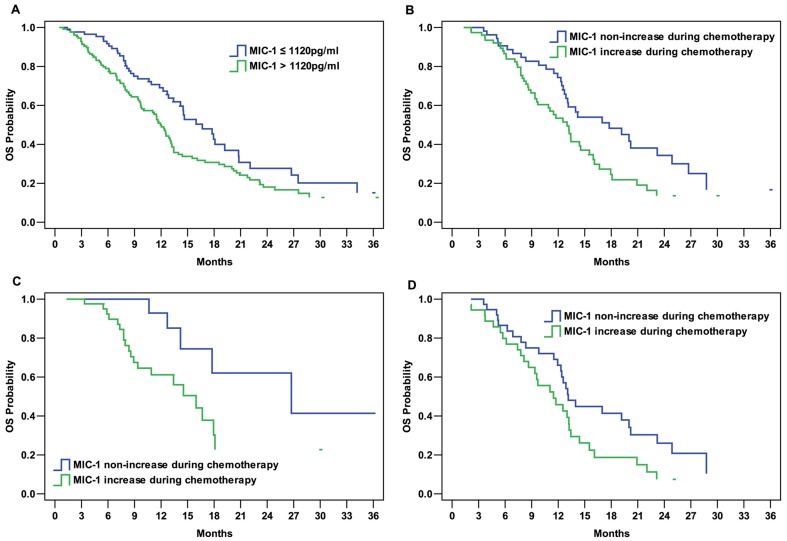
Kaplan–Meier curves of overall survival (OS) of patients according to macrophage inhibitory cytokine-1(MIC-1). A. OS curve of patients grouped by serum MIC-1 before chemotherapy (*P* = 0.015). B. OS curve of patients grouped by serum MIC-1 change during chemotherapy (*P* = 0.030). C. OS curve of patients with serum MIC-1 concentrations ≤1120 pg/ml before chemotherapy grouped by serum MIC-1 change during chemotherapy (*P* = 0.035). D. OS curve of patients with serum MIC-1 concentrations >1120 pg/ml before chemotherapy grouped by serum MIC-1 change during chemotherapy (*P* = 0.078).

In the 217 patients with MIC-1 concentrations before chemotherapy, the high MIC-1 concentration group (>1120 pg/ml) had shorter OS (11.9 months *vs.* 16.7 months; *P* = 0.015; Figure 3A) compared with patients with low MIC-1 concentrations (≤1120 pg/ml). However, the PFS did not significantly differ between the groups with high and low MIC-1 groups before chemotherapy (*P* = 0.443).

Of the 133 patients with MIC-1 data after chemotherapy, the OS was shorter in the MIC-1 increase group than in the non-increase group (13.0 months *vs.* 17.8 months; *P* = 0.030; Figure 3B). Similarly, the PFS did not significantly differ between these two groups (*P* = 0.900). Furthermore, among the patients with serum MIC-1 ≤1120 pg/ml before chemotherapy, patients exhibiting increase in MIC-1 concentration during chemotherapy had shorter OS than patients exhibiting no increase in the MIC-1 (16.0 months *vs.* 26.7 months; *P* = 0.035; Figure 3C). This trend was also found in the group of patients whose serum MIC-1 was >1120 pg/ml before chemotherapy, although the difference was not statistically significant (11.5 months *vs.* 13.1 months; *P* = 0.078; Figure 3D).

## Discussion

Several studies have previously reported that weight loss before chemotherapy is associated with poor survival in cancer patients, especially in patients with GC [3], [4], [19], [20]. The results of the present study also demonstrated that weight loss of >5% before chemotherapy predicted poor survival in patients with advanced GC.

Patients with gastrointestinal and lung cancers whose weight stabilized while on chemotherapy had significantly better PFS and OS than patients exhibiting continued weight loss [3], [20]. Hence, does weight loss during chemotherapy influence the outcomes in patients with advanced GC? Exactly how much weight loss is too much?

In the present study, four patterns in weight change were defined, and the results confirmed that the weight change pattern affected the treatment outcome. Patients exhibiting weight loss during chemotherapy had poor PFS and OS. If the weight loss was reversed during chemotherapy, the survival rates improved. This indicates that any method to prevent weight loss or prompt weight restoration could potentially improve the prognosis in these patients.

We then addressed the second question of how much weight loss is too much. Even weight loss of 5% alters measurable physiological parameters, such as immune response, results of lung and cardiac function tests, and autonomic regulation, and the response to chemotherapy may be altered by weight loss [21]. In the only study evaluating the significance of weight loss in a large group of patients receiving chemotherapy, it was reported that weight loss of even 5% before chemotherapy had a significant adverse effect on survival [22]. To investigate whether subtle weight loss of less than 5% was significant, we examined the effects of 1, 2, 3, and 4% weight loss on survival in the present study. Multivariate analysis indicated that >3% weight loss during chemotherapy was an independent prognostic indicator of poor OS. Therefore, >3% weight loss during chemotherapy in patients with advanced GC may alert the oncologist to a worse prognosis and the need for early interventions to address the weight loss.

Interventions to address weight loss in cancer patients, such as anti-inflammatory therapy [23]–[25], nutrition support [26]–[30] and exercise training [31], [32], have been investigated for a long time. Although some results have been encouraging, there is still lack of an adequate evidence base for its therapy [33].

MIC-1 was first identified as an appetite regulator when it was discovered that its overexpression in cancer and other diseases lead to anorexia/cachexia [17]. Johnen *et al*. [17] found that MIC-1 acts on TGF-β RII receptors in hypothalamic neurons, reduces neuropeptide Y expression, and increases pro-opiomelanocortin expression, which may decrease appetite. Overexpression of MIC-1/GDF15 in mice did not result in any reduction in energy expenditure as determined by indirect calorimetry, further highlighting the importance of reduced food intake to the associated reduction in body weight, adiposity and muscle mass. Furthermore, the study found that elevated serum MIC-1 concentrations were associated with significant weight loss that could be reversed by using antibodies to MIC-1, and serum MIC-1 concentrations were significantly associated with weight loss in patients with cachectic prostate cancer. Therefore, we hypothesized that high serum MIC-1 concentration can lead to weight loss by decreasing appetite in advanced GC patients. In the present study, the serum MIC-1 concentrations were higher in patients with advanced GC than in the controls. However, we failed to show a statistically significant relationship between MIC-1 concentrations and weight loss before or during chemotherapy. These findings are partly consistent with those reported in a previous study which showed no relationship between the MIC-1 serum concentration and nutritional status in patients with esophagogastric cancer [34]. Such results may be due to the complex additional factors that control food intake and perhaps because the circulating MIC-1 concentrations are simply not sufficiently elevated to overcome regulatory mechanisms and induce weight loss in advanced GC patients [34].

Some studies had also indicated that the serum MIC-1 concentration was closely associated with systemic inflammation [6], [34]. Although the exact mechanism by which systemic inflammation arises in cancer patients remains to be clarified, it is generally accepted that cancer-associated inflammation is modulated by cancer cells, host stromal cells, and their interactions [35]. C-reactive protein (CRP) is an acute-phase protein synthesized by hepatocytes, and its concentrations in the serum increase during inflammatory diseases [36]. Our study demonstrated that the serum MIC-1 concentration was significantly correlated with the serum CRP concentration, suggesting that MIC-1 might play a role in the etiology of systemic inflammation in advanced GC patients.

Elevated concentrations of circulating MIC-1 have also been reported to be associated with tumor grade, stage, and poor prognosis in some cancer patients [15], [34], [37]. Therefore, it needs to be ascertained whether the MIC-1 concentrations before and especially during chemotherapy have clinical significance in patients with advanced GC. In the present study, we found that both elevated serum MIC-1 concentrations before chemotherapy and increased serum MIC-1 concentrations during chemotherapy were independent prognostic factors of poor OS in patients with advanced GC, suggesting that MIC-1 might be a potential prognostic biomarker in these patients.

This exploratory study showed that weight loss during chemotherapy was associated with poor OS and that the survival rates improved if this weight loss was reversed during chemotherapy. MIC-1 did not correlate with weight loss; however, it was significantly correlated with systemic inflammation and OS, and might be a potential predictive and prognostic biomarker in patients with advanced GC. Future research should focus on the mechanism of weight loss during chemotherapy and interventions to ameliorate weight loss, which should be examined in prospective trials to assess the ability to improve the prognosis of these patients.
