# Correction: Change of Body Weight and Macrophage Inhibitory Cytokine-1 during Chemotherapy in Advanced Gastric Cancer: What Is Their Clinical Significance?

**DOI:** 10.1371/journal.pone.0097385

**Published:** 2014-05-07

**Authors:** The PLOS ONE Staff

The affiliation for the Academic Editor is incorrect.

The correct affiliation for Academic Editor Yuan-Jia Chen is: Department of Gastroenterology, Peking Union Medical College Hospital, Peking Union Medical College, Beijing, China

## References

[1] Lu Z, Yang L, Yu J, Lu M, Zhang X, et al (2014) Change of Body Weight and Macrophage Inhibitory Cytokine-1 during Chemotherapy in Advanced Gastric Cancer: What Is Their Clinical Significance? PLoS ONE 9(2): e88553 doi:10.1371/journal.pone.0088553 24586342 10.1371/journal.pone.0088553 PMC3938426

